# An evaluation of the Caribbean regulatory system centralized assessment process for medicines submitted 2017–2018 using the OpERA methodology

**DOI:** 10.1186/s40545-020-00261-z

**Published:** 2020-09-08

**Authors:** Lawrence Liberti, Rian Marie Extavour, Prisha Patel, Neil McAuslane

**Affiliations:** 1 Centre for Innovation in Regulatory Science, 160 Blackfriars Road, London, SE1 8EZ UK; 2 Caribbean Regulatory System, Caribbean Public Health Agency (CARPHA), 16-18 Jamaica Blvd., Federation Park, Port of Spain, Trinidad and Tobago

**Keywords:** Optimizing efficiencies in regulatory agencies (OpERA), Caribbean regulatory system (CRS), Caribbean community (CARICOM), Pan American health organization (PAHO)

## Abstract

**Background:**

The Caribbean Regulatory System is a centralized medicine assessment procedure established to serve the needs of the Member States of the CARICOM region. In order to better understand the effectiveness and efficiency of the processes implemented by the Caribbean Regulatory System for the regulatory assessment of medicines for the region, the system has been participating in the Optimizing Efficiencies in Regulatory Agencies (OpERA) program, a multinational endeavor to characterize the assessment procedures and the corollary metrics associated with medicine review activities in regulatory agencies and regional regulatory initiatives.

**Methods:**

The OpERA tool was used to collect process and specific milestone data for products approved by the Caribbean Regulatory System during 2017 (*n* = 10) and 2018 (*n* = 11).

**Results:**

The median total approval time was 57.5 days (25th/75th percentiles: 54, 60) in 2017 and 148 days (120, 163) in 2018. The median time to conduct the scientific assessment of the dossier was 37 days (24, 42) in 2017 and 66 (40, 132) days in 2018, within the target of 90 days for this activity. The time increases observed in 2018 were due to staff manpower limitations that reduced the ability of the system to conduct the timely assessment of applications. Based on these observations, recommendations to optimize the effectiveness and efficiency of the Caribbean Regulatory System include a commitment from Member States and partner organizations to the use of the procedure to accelerate product availability, encouraging the use of the Caribbean Regulatory System for non-generic products approved by a reference agency, ensuring the establishment of policy and legal frameworks to facilitate the rapid uptake of Caribbean Regulatory System registrations as marketing authorizations in the Member States, and maintaining the sustainability of the process through a fee-based approach.

**Conclusions:**

The observations obtained using the OpERA methodology indicate the Caribbean Regulatory System is an effective and efficient mechanism to provide recommendations to Member States for important medicines.

## Introduction

To maximize the use of limited resources, many agencies have evolved their processes to employ risk-based approaches, including reliance on prior reviews by reference agencies while also taking into consideration benefit-risk decisions based on local standards of care. According to the World Health Organization, reliance is a process whereby a regulatory authority in one jurisdiction may take into account/give significant weight to evaluations performed by another regulator or other trusted institution in reaching its own decision. In addition, factors such as the number of prior approvals and where approvals occurred, length of time on market, quality (similarity) of the product, local medical standard of care, and unmet medical need may contribute to the reliance decision. The relying authority remains responsible and accountable for decisions taken, even when it relies on the decisions and information of others.

These reliance approaches are supported by international regulatory convergence and alignment around guidelines such as those of the World Health Organization, International Council on Harmonization of Technical Requirements for Registration of Pharmaceuticals for Human Use (ICH), the Association of Southeast Asian Nations (ASEAN) and are underpinned by good regulatory practices [1–4]. Reliance has been recommended as an important efficiency tool for Latin America by the Pan American Health Organization (PAHO) [5]. The measurement of regulatory review performance should be documented and tracked to identify characteristics such as where time is spent, the input by the agency and the company, the number of review cycles and the outcomes, thus ensuring the efficiency of the review process as it evolves. Hence, the need for agencies to proactively and consistently measure their performance against stated target times is one of the World Health Organization (WHO) global benchmarking tool parameters [6].

### The Caribbean regulatory system

The Caribbean Regulatory System (CRS) is a regional reliance endeavor designed to benefit from new regulatory science approaches that improve efficiency [7]. From its inception in 2016, the CRS has functioned with one full-time technical officer or coordinator and has had various configurations of 1-2 part-time technical officers. The staff are registered pharmacists with knowledge of pharmacology, pharmaceutics, medicines information, therapeutics, and public health. The unit is funded by a grant from the Bill and Melinda Gates foundation, with an eye towards catalyzing a sustainable user-fee system in the future. The CRS has already begun charging user fees. The CRS is now resourced to hire two full-time staff: one as the technical coordinator and one as technical officer.

Agreed upon by Ministers of Health in 2014 to serve the needs of the CARICOM (Caribbean Community), the CRS is underpinned by the Caribbean Pharmaceutical Policy, which was adopted by the Ministers of Health in 2011 [8]. Located in Trinidad and Tobago within the Caribbean Public Health Agency (CARPHA), the CRS has been established as an initiative of the 15 Member States to support the timely and equitable access to essential medicines for the region’s 17 million inhabitants. The region consists of several small population Member States with limited regulatory and technical capacity needed to implement best/efficient practices (e.g., reliance mechanisms) [9, 10].

The CRS helps Member States perform key regulatory functions by using a reliance approach for market authorization that leverages decisions by reference authorities with a focus on essential medicines. Its work is intended to enable a sustainable enterprise in this resource-constrained environment. The CRS assists its Member States with the resource- and time-intensive task of evaluating medicines for safety, quality, and efficacy. All medicines reviewed by the CRS are required to have been approved by a designated reference authority. The list of reference NRAs is selected based on maturity and inclusion as a regional regulatory authority of reference by the Pan American Health Organization for the Americas. Once confirmed as eligible, the medicines intended for the CARICOM markets are verified as the same as in the original market and are then subject to an abridged review of key dossier elements. Sponsors are asked to submit English translations for documents that are not issued in English. However, where market authorization certificates are printed in Spanish, the CRS staff uses simple translation tools to convert it to English. Product information, including packaging and artworks, are required to be published in English for use in Anglophone countries. During dossier review, the following elements are verified: cover letter from the manufacturer/importer stating the product is the exact same as approved by the reference authority, market authorization granted by the reference authority, the product’s characteristics (e.g., formulation, packaging, indications), manufacturing sites, efficacy or bioequivalence, and safety reports (where applicable). Key documentation reviewed include the market authorization certificate or letter, the summary of product characteristics, certificates of compliance with Good Manufacturing Practices (GMPs), stability study data for the product under long-term conditions as per climatic Zone IVB, and under accelerated conditions, summaries of clinical trials or bioequivalence studies (where applicable), and periodic safety reports (where available). The review is conducted by dedicated, qualified reviewers and if favorable, the CRS recommends the product to National Regulatory Authorities (NRAs) and/or CARICOM Ministries of Health. These governments then determine whether to issue a sovereign marketing authorization. This aligns with CARPHA’s mandate to prevent diseases, promote health and respond to public health emergencies. The work of the CRS provides a platform for changing regulatory thinking, highlighting best practices, improving efficiencies for access to markets, and embracing new regulatory paradigms. In addition, the CRS helps CARICOM Member States with post-market surveillance and pharmacovigilance activities through a regional network, called VigiCarib.

Due to the voluntary nature of applicant participation in the CRS process, manufacturers determine the products to submit. However, the medicines should be listed on the WHO Essential Medicine List or be of public health value to the region. Since its inception, medicines with public health importance, such as anti-retrovirals and antibiotics, have been recommended by the CRS. Several medicines for the treatment of chronic non-communicable diseases (NCDs) have also been recommended, which will enable countries to access products to manage the growing burden of NCDs. The CRS recently recommended an innovative cure and an essential medicine to treat Hepatitis C that is not currently registered anywhere in the region along with a cholera vaccine and a biosimilar (pegfilgrastim). Applications or dossiers may be submitted to CARPHA/CRS directly or on request for review from one of the Member States. The latter route is a good option if a product has been in backlog and needs timely review.

### CIRS OpERA

To aid the CRS and other agencies to achieve goals of regulatory efficiency, The Centre for Innovation in Regulatory Science (CIRS) has developed a unique regulatory-strengthening program entitled OpERA: Optimizing Efficiencies in Regulatory Agencies. OpERA is a multi-year project initiated by CIRS in 2013 based on requests from regulatory agencies [11]. Objectives of the program are to (1) provide benchmarking data that can be used to define performance targets and focus ongoing performance improvement initiatives; (2) accurately compare the processes used in the review of new drug marketing authorizations; (3) encourage the sharing of information on common practices in order to learn from others’ experiences; and (4) encourage systematic measuring of the processes that occur during the review of new drug marketing authorizations [11].

The OpERA methodology comprises two components: a process assessment analysis designed to clearly assess the component activities associated with the medicine review and assessment processes within an agency or Regional Regulatory Initiative (RRI) and the collection of key milestone metrics aligned with the elements of the process assessment. The specific milestones identify time periods, review stages, and data points that have been selected by agencies and RRIs participating in the OpERA program so as to permit a detailed analysis of an agency’s efficiency. Results provide factual information that encourages adherence to processes that underlie the efficient review activities of each agency or RRI, defining and meeting regulatory review performance goals, improving review process efficiencies, and building a culture of self-measurement to encourage continuous process optimization. The program is designed to support the information needs of mature and maturing authorities through the use of performance metrics. The outcome of participation is the receipt of factual results that can be used to help better convey their mission and needs to policy-makers and other stakeholders as well as to continuously monitor their performance for purposes of improvement of timelines and quality of processes. The results generated by OpERA have been used by agencies to compare their activities against those of similar agencies and to provide the basis for a public discussion of new legislative approaches to the optimization of regulatory procedures [12–14].

With this background, the CRS agreed to participate in the OpERA program and provided both qualitative and quantitative information regarding its assessment process. Herein, we describe observations on the process of the CRS, derived from its participation in the OpERA program.

## Methods

### Process assessment

CIRS developed a standardized Regulatory Assessment Process Questionnaire that identifies specific assessment activities in five clusters: organization of the agency; types of review models; key milestones in the review process; Good Review Practices applied in the assessment and registration of medicines; and quality decision-making practices [15]. CIRS pre-filled the questionnaire with data from the public domain, after which staff of the CRS verified the information and completed the questionnaire.

### Product-specific metrics

The milestone dates were collected for each application in the study (Table 1). All time periods were measured in calendar days. In addition, qualitative data were requested for each product in order to characterize the application. These data included applicant name; whether the application was from a multinational or local company; the compound type; that is, new chemical entity, biological, or vaccine, the generic name or compound code; whether the compound was a WHO pre-qualified generic or vaccine; the trade name; the review type; that is, verification, abridged, or full; the general therapeutic class identified by ATC code (first-level anatomical group codes A-V) and whether it was a priority review. Applicant names, compound codes, and trade names could be masked for confidentiality. Using the OpERA tool, the following intervals were assessed:

- *Dossier validation and queue time:* The time between the date of receipt of the dossier and starting the scientific assessment
- *Scientific assessment time (total):* Time spent from the date of the start of the scientific assessment to the date of completion of all scientific assessments including both agency and applicant time
- *Applicant time (2017 only):* The time during which the review timing clock is stopped during the review while the agency awaits additional data requested from the applicant
- *Agency scientific assessment time (2017 only):* Total scientific assessment time minus the time a dossier is with an applicant
- *Applicant notification time*: The time from the completion of the scientific assessment to when the notification of final decision is sent to the applicant
- *Overall approval time:* The time from the date when the submission is received by the agency to when the notification of final decision is sent to the applicant

**Table 1:** Milestones and product characteristics collected through the OpERA program

Key milestone dates	
1a. Receipt of the dossier	
1b. Acceptance to file	
2a. Start of primary scientific assessment	
2b. Completion of primary scientific assessment	
3a. Primary assessment deficiency letter sent to applicant (if applicable)	
3b. Response from applicant (if applicable)	
4. Secondary assessment following deficiency letter response (if applicable)	
5. Succeeding Advisory Committee Review (if applicable)	
6. Completion of scientific assessment	
7. Marketing Authorization outcome: granted/rejected	
8. Final Acceptance by each Member State	

These data were then described using medians and percentiles to facilitate understanding of the variation around the median.

*Training and data collection parameters*: Following a series of CIRS-led WebEx training sessions, the central officer of the CRS completed the Regulatory Assessment Process Questionnaire and supplied datasets for products assessed by the CRS for the 2-year period from 1 January 2017 to 31 December 2018 using a secure password-protected bespoke data entry website. CIRS then validated the integrity of characteristics and milestone information, evaluated missing or non-conforming data, and worked with the CRS to resolve discrepancies. Descriptive statistics were used for numbers of products by category; medians and 25th/75th percentiles were calculated using Excel. CIRS then graphed the results using preset validated algorithms built into the proprietary OpERA system. A draft report was prepared and presented to the CRS to ensure appropriate interpretation of the observations.

### Observations

#### Process assessment

**Fig. 1:**
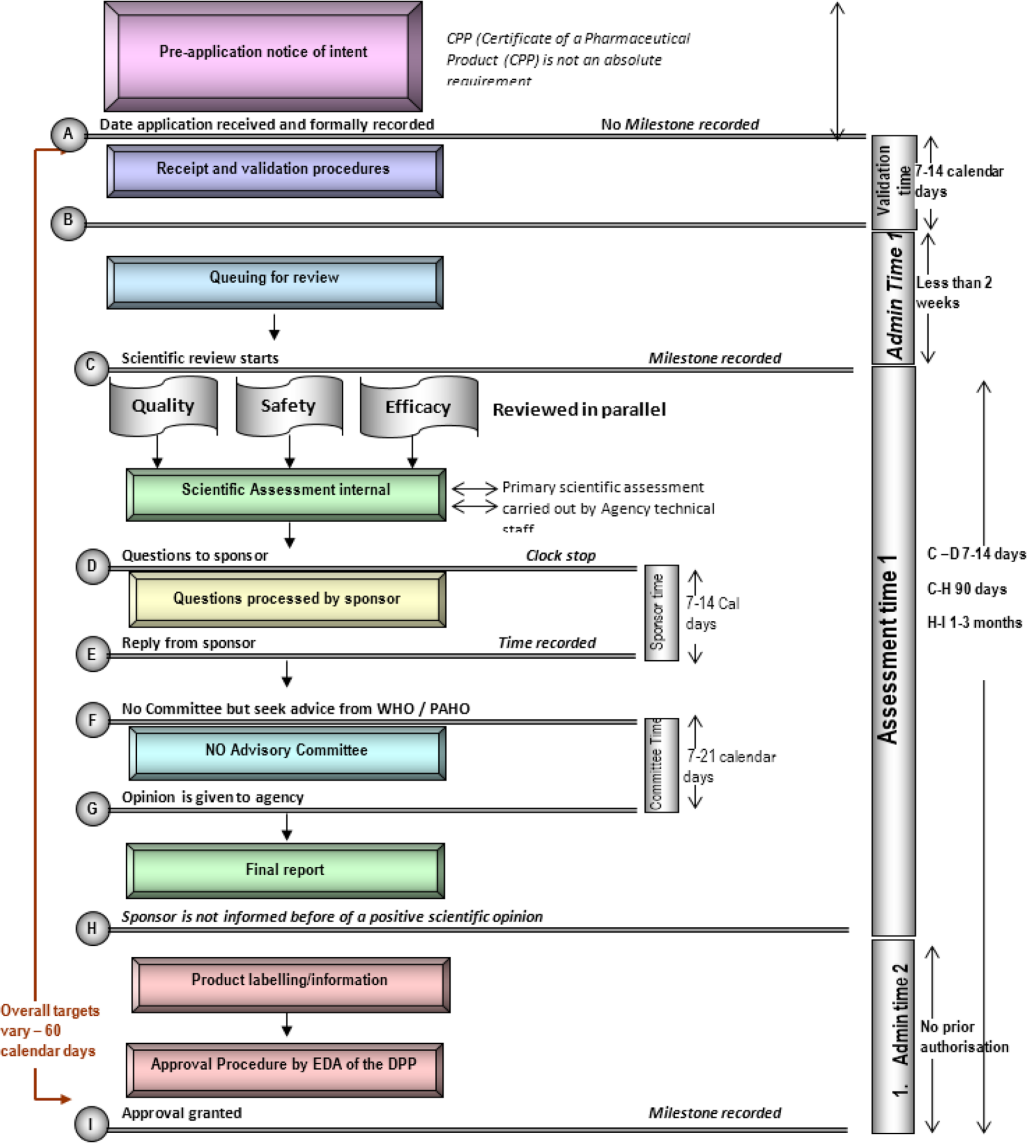
Flow chart of the regulatory assessment process employed by the CRS centralized procedure

The CRS staff completed the Regulatory Assessment Process Questionnaire with CIRS in June 2017. Based on information provided, the following key observations about the system were made. Any products for which the CRS undertakes a review must have received a prior approval by a reference authority designated by the CRS **(**EMA, US FDA, Health Canada, WHO-Prequalification, ANMAT (Argentina), ANVISA (Brazil), ISP (Chile), INVIMA (Colombia), CECMED (Cuba), or COFEPRIS (Mexico). The process by which the CRS conducts its review is illustrated in Fig. 1. The CRS conducts a verification review of the product’s quality, safety, and efficacy presented in the dossier based on a similar procedure applied by the WHO Pre-qualification (PQ) program [16]. The CRS does not require a Certificate of Pharmaceutical Product (CPP) before an application is accepted. However, other documentation and evidence of authorisation issued are accepted in place of the CPP. These include full market authorization, electronic samples (including artworks and labels), and confirmation of the quality of manufacturing via certificates of Good Manufacturing Practices issued by the reference authority. It is not necessary for an application to be legalized by an Embassy or Consulate.

The target time for the scientific assessment is within 90 days. Questions to the applicant trigger a clock stop, and applicants are requested to respond to information requests within 14 days. Following a positive recommendation by the CRS, CARPHA issues a certificate recommending the product for marketing authorization in Member States. A member state is expected to review the decision and accept or reject it for its jurisdiction within 60 days of notification.

#### Product-specific metrics for 2017

During 2017, 14 products were submitted to the CRS for assessment of which 10 (from 2 applicants) received positive recommendations. Two recommendations had decisions that were being re-assessed by the applicant, and two remained in the assessment queue. All 14 products were generics, of which 12 were WHO-PQ generics. None of the applications were submitted by local companies. Twelve of the products were anti-infectives and 2 were oncology products.

**Fig. 2:**
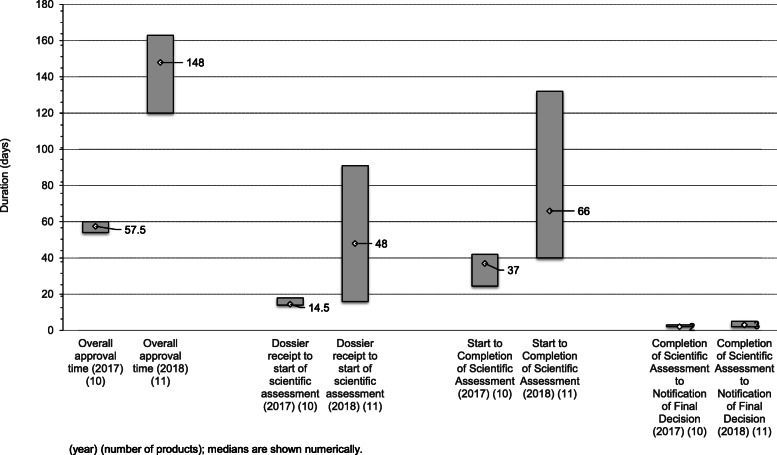
Comparison of key activities for 2017 and 2018 (medians with 25th–75th percentiles)

The median overall approval time for all products approved in 2017 was 57.5 days (25th and 75th percentile of 54 and 60 calendar days, respectively). This comprised a median of 14.5 days queue time (from dossier receipt to start of scientific assessment), 37 days for the scientific assessment phase, and 2 days from the completion of the assessment to the notification of the applicant of the decision. The 37 days for the scientific assessment comprised a median of 32 days of agency time and 6 days of company time (Fig. 2).

#### Product-specific metrics for 2018

During 2018, 11 products were approved by the CRS. Three of these were submitted in 2017 and 8 in 2018. These 11 products comprised 3 pre-qualified generics, 6 generics, and 2 vaccines. The most common therapeutic areas were cardiovascular and anti-infectives (2 products each); 2 products were cholera vaccines. All 11 products were submitted by international companies.

The median overall approval time for all products approved in 2018 was 148 days (25th and 75th percentile of 120 and 163 calendar days, respectively). This comprised a median of 48 days queue time (from dossier receipt to start of scientific assessment), 66 days for the scientific assessment phase, and 3 days from the completion of the assessment to the notification of the applicant of the decision. The 66 days for the scientific assessment comprised both agency and applicant time.

The CRS has set its target time for Scientific Assessment at 90 days. We observed that during 2017, all 10 of the products had a Scientific Assessment time of less than 90 days. For the 2018 cohort of 11 products, 7 (64%) were faster than the target with the remaining 4 (36%) taking longer than 90 days.

#### Year-on-year comparisons

Figure 2 compares the key metrics for 2017 and 2018. Although the total number of approvals was similar between the years, the overall median approval time increased from 57.5 to 148 days. This was influenced by a more than 3-fold increase in the median queue time from 14.5 to 48 days together with an increase in median scientific assessment time from 37 to 66 days.

## Discussion

This is the first multi-year comprehensive analysis of the regulatory assessment activities being undertaken by the CRS. The CRS is an important model for emerging regulators. It uses a dedicated, lean review staff that provides fit-for-purpose reviews of essential medicines in a region that has been challenged to provide equitable access to quality medicines recognized to be safe and effective therapies for important illnesses within the jurisdictions.

Although the time for scientific assessment increased from 2017 to 2018 due to staffing issues at the CRS for part of the year, the median scientific assessment time in 2018 (66 days) was well below the target 90 days established by the CRS. The increase in assessment time may have been a result of staff transitions and a change in the types of applications received. In 2017, staff consisted of one full-time technical officer, but later that year the office became vacant. As a result, there was a period of transition after the position became vacant in December 2017 until March 2018 when a new full-time officer was employed and trained. During that time, the work was done by a part-time technical officer. In addition, in 2018 the CRS received fewer applications for products from the WHO pre-qualification program for the Collaborative Procedure. However, more applications for products approved by CRS reference regulatory authorities were received that required a new process of verification review and additional time. Further, we observed that during the second half of 2018, queue and assessment times decreased to 2017 levels. No data regarding uptake by individual Member States were available to the authors at the time of this publication.

The overall results shown here may not reflect the true efficiency of the CRS for several reasons. During 2017, the CRS undertook its first product assessments. Good Review Practices were being instituted and systems were being refined during this period. It can take several years for such practices to be well integrated into a regulatory authority’s procedures. During 2018, the nature of the applications to the CRS changed from predominantly WHO pre-qualified products through the WHO Collaborative Procedure to Reference Authority-approved products. This necessitated developing more extensive verification procedures including using public sources of information on reference authority websites for verification. Reference authorities did not share confidential information on these products. Another time-consuming aspect for the reviewers included the receipt of dossier submissions of poor quality due to absence of documentation of approvals by a recognized reference authority, with incomplete information, resulting in rejections because of ineligibility for review.

Between 2017 and 2018, the CRS received submissions of products that were pre-qualified by the WHO. As a result, these products were primarily generic and non-biologic. In November 2019, the CRS introduced a procedure to review biologic products, based on the WHO’s procedure for review of similar biotherapeutic products (June 2018). With this new pathway, the CRS is expected to receive dossiers for biologic products, which would facilitate access to cheaper therapies for cancer treatment in the region.

Supported by the OpERA-based observations for 2017 and 2018, we believe the following activities could contribute to optimizing the value and effectiveness of the CRS:

1. The Member States should commit to consistent staffing of the assessment team. This is dependent on the teamwork and collaboration of key stakeholders, including CARICOM and CARPHA, in the implementing roles, and PAHO, in the technical support role. External partners are also essential for the success of the CRS, not only through financing, but through training and mentorship as well.
2. Applications for non-generic products approved by a reference agency should be encouraged.

The 60-day target timeline for review of the CRS decision by a Member State should be adhered to by participating agencies. This requires integration with the establishment of policy and legal frameworks to facilitate the rapid uptake of CRS registrations as marketing authorizations in the Member States, supported by CARPHA’s legal authority.

Furthermore, to maintain the sustainability of the CRS staff and process, a fee-based approach has been recommended. In October 2019, the CRS announced that it will begin charging modest user fees to the companies that apply for medicines recommendation through the CRS, starting in November 2019 (manufacturers: $300 USD per medicine application; local importers: $150 USD per medicine application). The “applicant” is responsible for paying the application fee. This fee is due before the product can be considered, and will be subject to a renewal fee at the time of expiry of the recommendation. Applicants must provide all requested documentation within one year of initial submission, or re-apply by paying the application fee again. These fees are designed to cover the costs of staff at CARPHA with mechanisms that ensure reliable funding streams, with contributions from Member States and product-based fees from the applicants [17].

The CRS is faced with the challenge of serving the needs of Member States that have limited regulatory competencies. An analysis conducted by PAHO found that the non­Latin Caribbean lags significantly in terms of having in place the 20 key indicators for regulatory capacity having only implemented 39% of the basic indicators [9]. Specifically, basic indicator data show poor capacity in core functions such as marketing authorization, pharmacovigilance, and post­market surveillance. Only 55% have a legal provision requiring marketing authorization of pharmaceutical products (“registration”), and an internal PAHO analysis found that full implementation of marketing authorization procedures for generic medicines ranges from 0 to 25% in some Caribbean countries [9]. These challenges are faced by other developing economies, so the experience of the CRS and the recommendations above can serve as a model for an efficient, centralized approach to the regulation of necessary therapeutics. A main barrier to this process is that larger reference authorities, due to a variety of factors including heavy workloads, do not prioritize confidentiality agreements with very small regulatory authorities, where there may not be very much mutual benefit in information sharing. Additional barriers include the need for liaisons who speak English and who may assist to confirm undocumented information or verify missing information and the terms of established confidentiality agreements between the NRA and the manufacturer, where the NRA is not allowed to disclose aspects of assessments or inspections.

Applicants may be asked to share the final assessments issued by the reference agency, but this is discretionary.

## Conclusions

Based on the observations obtained using the OpERA methodology, we have found the CRS is an effective and efficient mechanism that focuses on the key elements required to assure that quality, safe, and effective medicines can be recommended to CARICOM Member States. Furthermore, these procedures are undertaken efficiently within time frames that do not unduly impede recommendations to the Member States. The data from this study have provided the CRS with a baseline against which assessments of the impact of process improvement initiatives can be measured, process efficiency comparisons can be made over time, and experiences can be shared with other regions facing similar regulatory challenges.

## Abbreviations

ASEAN: Association of Southeast Asian Nations
CARICOM: Caribbean Community
CARPHA: Caribbean Public Health Agency
CPP: Certificate of Pharmaceutical Product
CIRS: Centre for Innovation in Regulatory Science
CRS: Caribbean Regulatory System
ICH: International Council on Harmonization of Technical Requirements for Registration of Pharmaceuticals for Human Use
NCD: Non-communicable disease
NRA: National Regulatory Authority
OpERA: Optimizing Efficiencies in Regulatory Agencies
PAHO: Pan American Health Organization
PQ: Pre-qualification
RRI: regional regulatory initiative
WHO: World Health Organization

## References

[1] Choong SSF, Lim JCW, Tominaga T (2018). Developing key performance indicators to measure the progress of regional regulatory convergence and cooperation in Asia-Pacific economic cooperation (APEC). AAPS Open.

[2] Liberti L, Breckenridge A, Hoekman J (2016). Accelerating access to new medicines: current status of facilitated regulatory pathways used by emerging regulatory authorities. J Pub Health Pol.

[3] Luigetti R, Bachmann P, Cooke E, Salmonson S (2016). Regulatory collaboration, collaboration, not competition: developing new reliance models. WHO Drug Info.

[4] World Health Organization. Good regulatory practices: guidelines for national regulatory authorities for medical products https://www.who.int/medicines/areas/quality_safety/quality_assurance/GoodRegulatory_PracticesPublicConsult.pdf. Accessed 12 January 2020.

[5] Freitas, M. PAHO – Regulatory reliance principles: concept note and recommendations. 11:36, 17 de September de 2019. http://prais.paho.org/en/paho-regulatory-reliance-principles-concept-note-and-recommendations/ Accessed 12 January 12, 2020.

[6] World Health Organization. WHO Global Benchmarking Tool (GBT) for evaluation of national regulatory systems. https://www.who.int/medicines/regulation/benchmarking_tool/en/ Accessed 3July 2019.

[7] Preston C, Freitas Dias M, Peña J, Pombo ML, Porrás A (2020). Addressing the challenges of regulatory systems strengthening in small states. BMJ Glob Health.

[8] CARPHA. http://new.carpha.org/Portals/0/Documents/Caribbean_Pharmaceutical_Policy-2013.pdf Accessed 8 January 8 2020.

[9] Pan American Health Organization. National Regulatory System: organizational structure and legal basis, and the provisions for medicines regulation in the Americas. PRAIS — regional platform on access and innovation for health technologies. Washington: PAHO; PRAIS Bull 2015;2(1).

[10] Preston C, Chahal HS, Porrás A, Cargill L, Hinds M, Olowokure B (2016). Regionalization as an approach to regulatory systems strengthening: a case study in CARICOM member states. Rev Panam Salud Publica.

[11] Rodier C, Patel P, McAuslane N, Liberti L: CIRS R&D Briefing 74: The OpERA programme: Measuring process and performance in regulatory agencies. January 2020. Centre for Innovation in Regulatory Science. London, UK. accessed at: https://www.cirsci.org/publications/cirs-rd-briefing-74-opera-programme/.

[12] Hashan H, Aljuffali I, Patel P, Walker S. The Saudi Arabia Food and Drug Authority: An Evaluation of the Registration Process and Good Review Practices in Saudi Arabia in Comparison with Australia, Canada and Singapore. Pharm Med. 2015. https://doi.org.10.1007/s40290-015-0124-4. 10.1007/s40290-015-0124-4 PMC4718932 26834481

[13] Mashaki Ceyhan E, Gürsöz H, Alkan A, Coşkun H, Koyuncu O, Walker S. The Turkish Medicines and Medical Devices Agency: Comparison of Its Registration Process with Australia, Canada, Saudi Arabia, and Singapore. Front Pharmacol. 2018. doi.org/10.3389/fphar.2018.00009. 10.3389/fphar.2018.00009 PMC5789679 29422861

[14] Keyter A, Salek S, Banoo S, Walker S. The south African medicines control council: comparison of its registration process with Australia, Canada, Singapore, and Switzerland. Front Pharmacol 2019 https://doi.org/10.3389/fphar.2019.00228. 10.3389/fphar.2019.00228 PMC6426768 30923501

[15] McAuslane N, Cone M, Collins J, Walker S (2009). Emerging markets and emerging agencies: a comparative study of how key regulatory agencies in Asia, Latin America, the Middle East and Africa are developing regulatory processes and review models for new medicinal products. Drug Inform J.

[16] World Health Organization. The WHO prequalification programme and the medicines patent pool: a primer. 2011. http://apps.who.int/prequal/info_general/documents/FAQ/PQ_PatentPool.pdf Accessed 12 January 2020.

[17] CARPHA. http://new.carpha.org/What-We-Do/Programmes-and-Projects/CRS/Operational-Policy Accessed 12 January 2020.

